# Edematous Ileocecal Valve Mimicking Incomplete Reduction after Intussusception

**DOI:** 10.5334/jbsr.3281

**Published:** 2023-10-02

**Authors:** Frédéric Marie Quatannens, Michael Aertsen, Stéphanie Braspenningx

**Affiliations:** 1 KU Leuven, BE; 2 Katholieke Universiteit Leuven Faculteit Geneeskunde, Universitaire Ziekenhuizen Leuven, Universiteit Hasselt, BE

**Keywords:** intussusception, ileocecal valve edema, single contrast enema

## Abstract

**Teaching Point:** An edematous ileocecal valve may mimic a residual intussusception after reduction. Differential diagnosis is important for therapeutic implications.

## Case History

**Figure 1:**
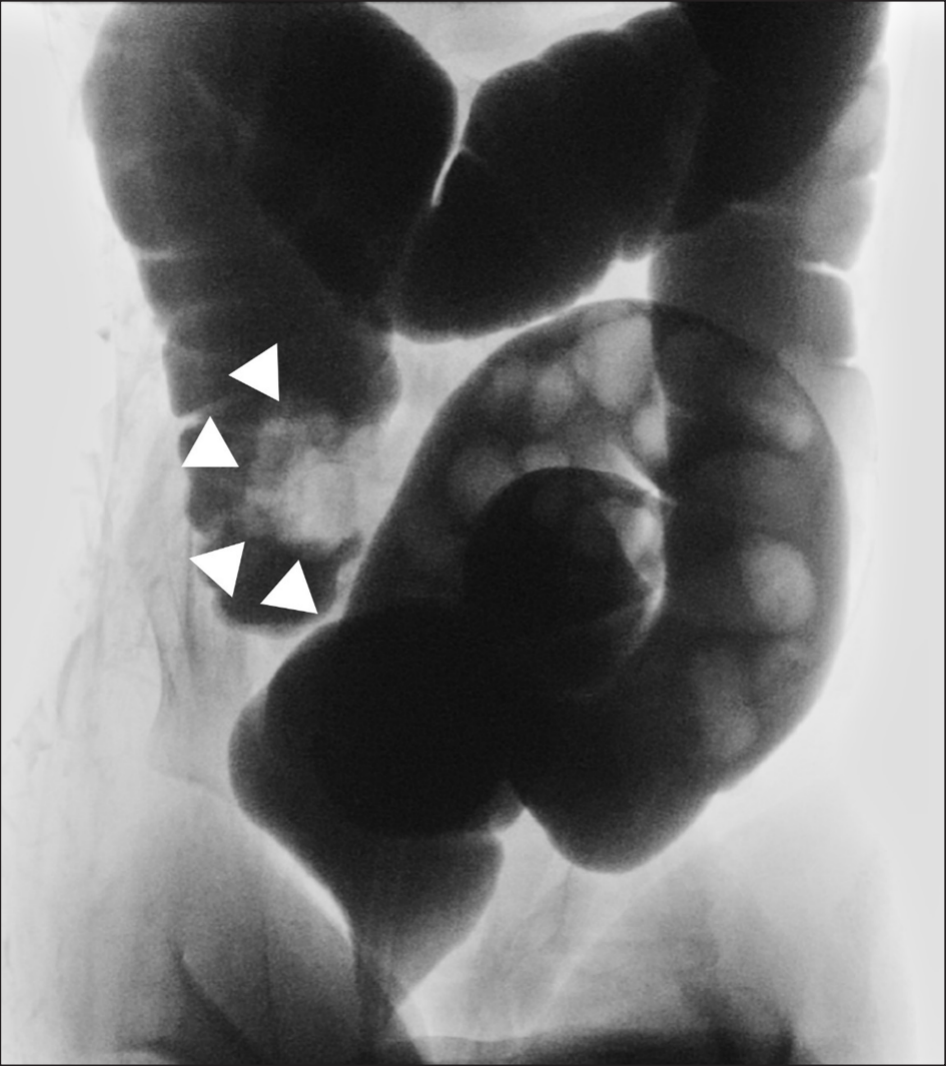


A two-year-old boy was transferred to the emergency department from a regional hospital with intermittent episodes of intense abdominal pain. An intussusception was diagnosed by ultrasound whereafter hydrostatic reduction with single-contrast enema was attempted. The reduction was diagnosed incomplete (Figure 1), and the child was transferred to a tertiary center.

**Figure 2:**
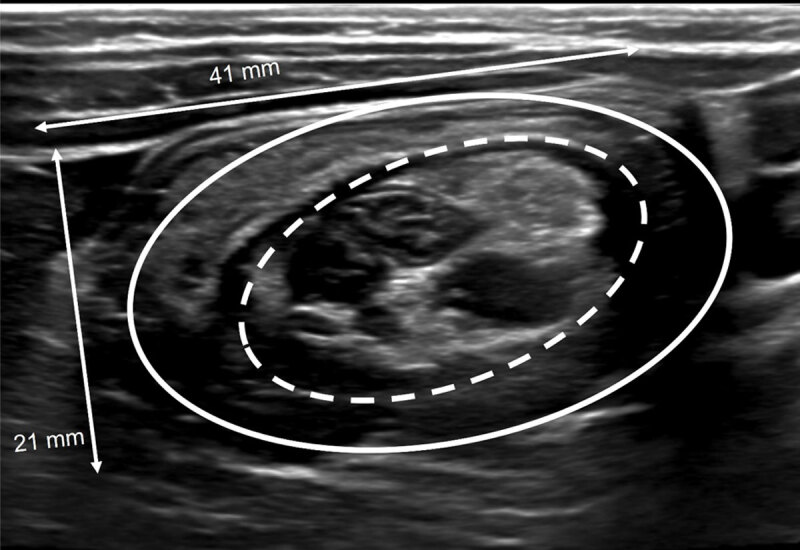


**Figure 3:**
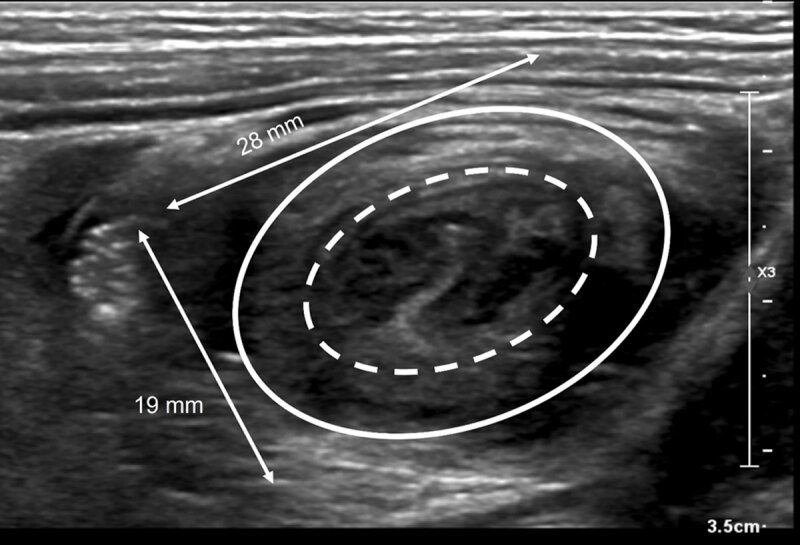


Abdominal ultrasound showed a small target sign in the transverse plane without presence of mesenterial fat, vessels or lymph nodes between the layers of the target sign (Figure 2). The width of the presumed intussusception was 25 mm over a short segment of 16 mm. The cecum was oriented as normal, and the appendix was in a normal position. In retrospect this correlated with the external single-contrast enema images (Figure 1). The patient had a second episode of ileocolic invagination within 12 hours, which was again reduced with hydrostatic reduction under fluoroscopy (Figure 3). After this second reduction, he was administered for observation and normal intake was resumed the next day, without recurrence. Follow-up ultrasound 48 hours after the first episode demonstrated an important reduction in ileocecal valve size and the patient was discharged after 48 hours.

## Comments

Ileocolic intussusception is one of the most common pediatric abdominal emergencies. Fluoroscopic enema reduction (air- or water-soluble contrast) is the initial treatment of choice with a success rate of 80–95%, sonographic-guided hydrostatic saline enema reduction has been shown a good alternative. Operative reduction is the alternative in patients with complications or multiple failed reduction attempts [1].

After fluoroscopic attempted reduction of an ileocolic intussusception, a persistent intraluminal filling defect may be seen in the cecum, posing a diagnostic dilemma in differentiating a residual or recurrent intussusception, a pathological lead point or a residual edematous ileocecal valve. Post-reduction sonography may solve this dilemma showing a ‘smaller’ donut/target sign (Figure 2) compared with the pre-reduction lesion (Figure 3) and the lack of invaginated mesentery and lymph nodes. Patency may be confirmed by visualization of continuous air bubbles between the cecum and terminal ileum. Correct interpretation of intussusception and the completeness of reduction is important given the therapeutic and clinical consequences [1].
